# Multi-host model and threshold of intermediate host *Oncomelania* snail density for eliminating schistosomiasis transmission in China

**DOI:** 10.1038/srep31089

**Published:** 2016-08-18

**Authors:** Yi-Biao Zhou, Yue Chen, Song Liang, Xiu-Xia Song, Geng-Xin Chen, Zhong He, Bin Cai, Wu-Li Yihuo, Zong-Gui He, Qing-Wu Jiang

**Affiliations:** 1 Department of Epidemiology, School of Public Health, Fudan University, Shanghai 200032, China; 2 Key Laboratory of Public Health Safety, Ministry of Education (Fudan University), Shanghai 200032, China; 3 Tropical Disease Research Center, Fudan University, Shanghai 200032, China; 4 School of Epidemiology, Public Health and Preventive Medicine, Faculty of Medicine, University of Ottawa, Ottawa, K1H 8M5, Canada; 5 Department of Environmental and Global Health, College of Public Health and Health Professions, University of Florida, Gainesville, FL 32611, USA; 6 Guichi Anti-Schistosomiasis Station, Anhui 247100, China; 7 Junshan Anti-Schistosomiasis Station, Hunan 414000, China; 8 Puge Center for Disease Prevention and Control, Sichuan 615300, China

## Abstract

Schistosomiasis remains a serious public health issue in many tropical countries, with more than 700 million people at risk of infection. In China, a national integrated control strategy, aiming at blocking its transmission, has been carried out throughout endemic areas since 2005. A longitudinal study was conducted to determine the effects of different intervention measures on the transmission dynamics of *S. japonicum* in three study areas and the data were analyzed using a multi-host model. The multi-host model was also used to estimate the threshold of *Oncomelania* snail density for interrupting schistosomiasis transmission based on the longitudinal data as well as data from the national surveillance system for schistosomiasis. The data showed a continuous decline in the risk of human infection and the multi-host model fit the data well. The 25th, 50th and 75th percentiles, and the mean of estimated thresholds of *Oncomelania* snail density below which the schistosomiasis transmission cannot be sustained were 0.006, 0.009, 0.028 and 0.020 snails/0.11 m^2^, respectively. The study results could help develop specific strategies of schistosomiasis control and elimination tailored to the local situation for each endemic area.

Schistosomiasis, a major neglected tropical disease, affects approximately 240 million people worldwide with more than 70 million disability-adjusted life years, and there are more than 700 million people living in endemic areas^1^,^2^,^3^. Both the London Declaration on Neglected Tropical Diseases^4^ and the World Health Organization (WHO) 2020 Road Map^5^ aim to substantially reduce the global burden of neglected tropical diseases and one of the proposed goals is the regional elimination of schistosomiasis in the Americas and Western Pacific Region and the country elimination of schistosomiasis in selected African countries by 2020^5^. China has a new goal to eliminate schistosomiasis as a public health threat from the whole country by the 2016–2025 period^6^.

*Schistosoma japonicum*, one of schistosomes infecting humans, is prevalent in the Western Pacific Region (e.g., China and the Philippines), and is widely recognized as a zoonosis. It is a multi-host parasite with water buffalo being the primary host. Over the past six decades, China has made a great achievement in controlling schistosomiasis, eliminating transmission of *S. japonicum* in 5 provinces and reducing transmission intensities in the remaining 7 endemic provinces^7^. Currently, an integrated control strategy, assuming that a reduction in transmission of *S. japonicum* from definitive hosts (human and bovine) to the intermediate *Oncomelania* snail host would eventually block the transmission of the parasite, has been carried out throughout endemic areas in China since 2005^7^,^8^,^9^. The prevalence of human infections was reduced to 1% in some endemic regions^8^,^10^, but remained as high as 3% in other endemic areas^9^,^10^. The transmission of *S. japonicum* is relatively complicated, and there has been a debate on the universal suitability of the integrated strategy to control and eventually interrupt schistosomiasis transmission^11^. Beside human and bovine, more than 40 species of wild and domesticated mammals can serve as potential zoonotic reservoir hosts for *S. japonicum*^7^. In addition, there are also other factors such as low sensitivity/specificity of current diagnostic tools for infections and praziquantel treatment failures, impacting on the progress towards elimination of transmission of *S. japonicum*^7^,^12^.

Mathematical models are an important tool for predicting the transmission dynamics of a neglected tropical disease and provide critical insights to decision makers to develop and improve strategies of disease control and elimination. Macdonald^13^ and Hairston^14^ developed two mathematical models for the transmission dynamics of schistosomes as early as 1965, and since then, there have been a number of other models^15^,^16^,^17^,^18^,^19^,^20^,^21^,^22^,^23^,^24^. Williams *et al*.^19^ extended Barbour’s classic (1996)^18^ model to study the transmission of *S. japonicum*, and this extended two-host (i.e., human and bovine) model was tested for its validity^25^,^26^. However, in a five-year longitudinal study conducted in Anhui province of China, Zhou *et al*.^9^ found that the extended two-host model fitted the field observations of the first 3 years well but did not fit the data of the years 4 and 5. One important reason might be that the model did not take other important livestock or animals into consideration^27^,^28^.

*Oncomelania hupensis* snail is the only intermediate host for *S. japonicum*, and therefore its control is one of the key measures to eliminate the transmission of *S. japonicum*. Chemical molluscicides and environmental management targeting entire snail habitats have been the major intervention measures that lead to the elimination of *S. japonicum* transmission in 5 provinces in China^7^,^29^,^30^. An important question has been raised whether there is a ‘breakpoint’ (threshold) for *Oncomelania* snail population density concerning the risk of human infection. Understanding the behavior of the host-schistosome system in the vicinity of the transmission breakpoints remains a priority area of research^31^. It is also important to determine when interventions can be safely stopped^32^. In this study, we extended the multi-host model on the basis of the Williams’ two-host model^33^ to assess and predict the effectiveness of different interventions on the transmission dynamics of *S. japonicum* and to estimate the threshold value of *Oncomelania* snail density for interrupting the transmission of *S. japonicum*.

## Results

### Prevalence of infections, density of snail population and model prediction

**Figure 1:**
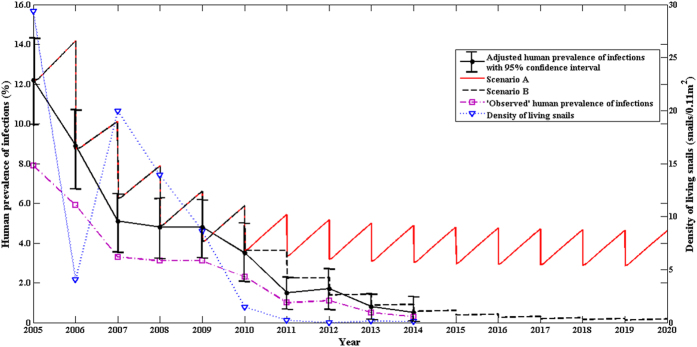
Effects of the comprehensive control strategy on human prevalence and density of living snails at Guifang village. Two control scenarios are modeled here, Scenario A represented the integrated control strategy including human chemotherapy, bovine removal, improvement in sanitation, health education, and environmental modification; Scenario B included the control measures implemented in scenario A and modifying the most of marshlands with snails by agricultural practice since 2010.

We calculated the following prevalences of human infection: 1) the “observed” prevalence is the proportion of individuals with positive stool examination; 2) the adjusted prevalence is the “observed” prevalence of human infection after taking the sensitivity and specificity of the Kato-Katz method into consideration; and 3) the multi-host model provides a modeled prevalence of human infection based on control strategies implemented. Figure 1 presents the prevalences and density of living snails at Guifang village over the 15-year period under the two control strategies (i.e., Scenarios A and B) (Fig. 1). The modeled prevalence showed a good agreement with the adjusted prevalence (no statistical difference between the two) and both had a sharp drop from 2005 to 2007, but no notable changes from 2007 to 2009 regardless further reduction in the density of living snails. From 2009 to 2011, the data showed a further sharp decline in the prevalence of human infection (Fig. 1). The density of living snails decreased from 29.35 (snails/0.11 m^2^) in 2005 to 0 (snails/0.11 m^2^) in 2012, and remained low at the level of approximately 0.1 (snails/0.11 m^2^) afterwards (Fig. 1).

**Figure 2:**
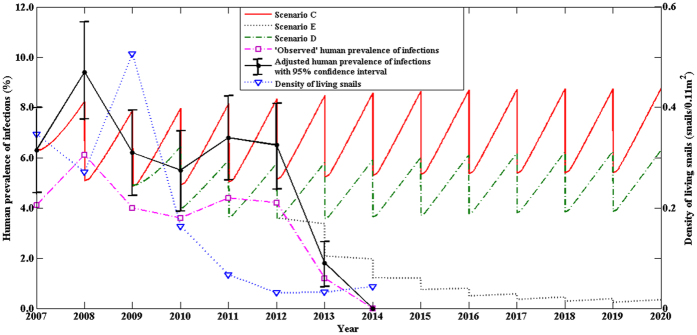
Effects of the integrated control strategy on human prevalence and density of snails at Changjang village. Three control scenarios are modeled here, Scenario C represented the control strategy including concurrent chemotherapy of humans and bovines, sanitation, environmental modification, snail control by molluscicide, and health education; Scenario D included the control measures carried out in Scenario C, establishing safe grazing pastures and fencing pastures since 2010; Scenario E included the measures implemented in Scenario D and bovine removal since 2013.

Figure 2 shows the prevalences and density of living snails at the village of Changjiang, over the 13-year period under the three control strategies (i.e., Scenarios C, D, and E). The prevalence of human infection showed no obvious change from 2007 to 2012 except for the year of 2010, and then presented a sharp drop from 2012 to 2014 (Fig. 2). The density of living snails fluctuated substantially between 2007 and 2009, then showed a sharp drop from 2009 to 2012, and became stable afterwards at the level of approximately 0.03 or 0.04 (snails/0.11 m^2^) (Fig. 2).

**Figure 3:**
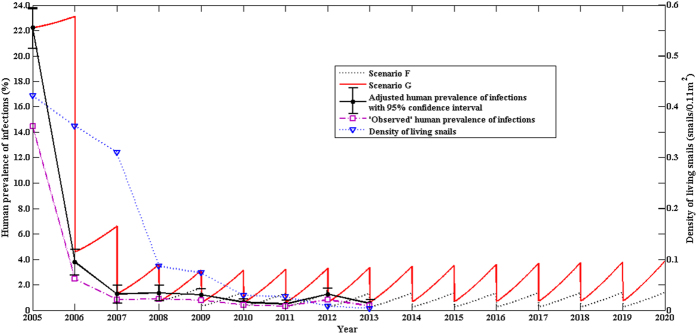
Effects of the comprehensive control strategy on human prevalence and density of living snails in Tezi town. Two control scenarios are modeled here, Scenario F represented the control strategy including concurrent chemotherapy of humans and bovines, sanitation, environmental modification, snail control by molluscicide, health education, establishing safe grazing pastures, and prohibiting pastures in the snail-infested grasslands; Scenario G included the control measures implemented in Scenario F and treating all snail habitats of the town by repeated mollusciciding and environmental management since 2008.

Figure 3 presents the prevalences of human infection and density of living snails in the town of Tezi over the 15-year period under the two control strategies (Scenarios F and G). Again, the modeled prevalence was very close to the adjusted prevalence with no statistically significant difference (Fig. 3). The prevalence of human infection showed a sharp drop from 2005 to 2007, and remained low afterwards, and so was the density of living snails (Fig. 3).

### Correlation between prevalence of human infection and density of living snails

There was a significant correlation of adjusted prevalence of human infection with the snail density at the village of Guifang (*r* = 0.85, *P* = 0.002) and in the town of Tezi (*r* = 0.85, *P* = 0.004); however, there was no such a significant correlation at the village of Changjiang (*r* = 0.21, *P *= 0.610).

### Threshold of *Oncomelania* snails

**Figure 4:**
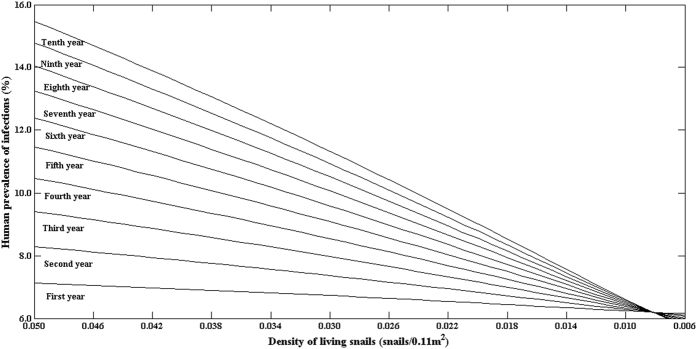
10-year predicted human prevalence of infections under different densities of living snails using the data from Changjiang village in 2009.

Data from the 8 national surveillance sites and the 3 fields were used to estimate the threshold values of *Oncomelania* living snail density for interrupting the transmission of *S. japonicum*, and a total of 59 estimated threshold values of *Oncomelania* snails were obtained. Figure 4 presents the changes in the predicted prevalence of human infection with calendar year under different snail densities based on data from the village of Changjiang village, Hunan province, in 2009. It was estimated that the threshold value of *Oncomelania* snail density was 0.0082 snail/0.11 m^2^ for interrupting the transmission of *S. japonicum*. When the density of living snails is less than 0.0082 snails/0.11 m^2^, the predicted prevalence of human infection decreases with calendar year independent of interventions. On the contrary, when the density of living snails is above the estimated threshold value and no intervention is carried out, the prevalence of human infection increases with calendar year after the snail density accounted for (Fig. 4).

**Figure 5:**
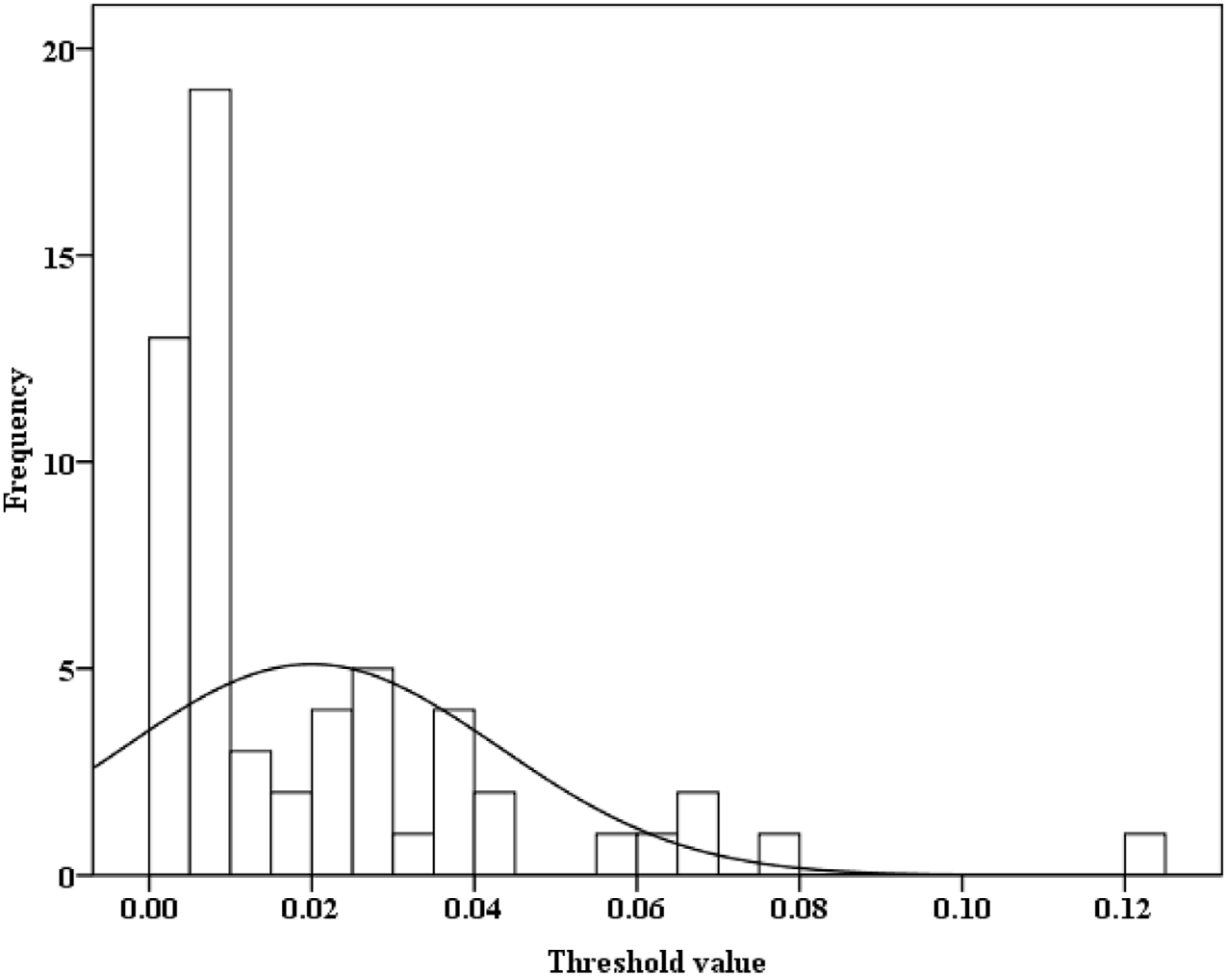
A histogram of the threshold values (snails/0.11 m^2^) of *Oncomelania* snail density for interrupting the transmission of *S. japonicum*.

Figure 5 presents 59 estimated threshold values of *Oncomelania* snail density for various endemic regions. The distribution of the estimated threshold values was positively skewed (Kolmogorov-Smirnov Z = 1.667, *P* = 0.008) (Fig. 5). The range of the estimated threshold was from 0.0005 snails/0.11 m^2^ to 0.12 snails/0.11 m^2^, and most of these estimated threshold values were low. The 25^th^, 50^th^ (median), and 75^th^ percentiles, and mean of the estimated threshold values were 0.006, 0.009, 0.028 and 0.020 snail/0.11 m^2^, respectively.

## Discussion

*S. japonicum* is a zoonotic parasite, and more than 40 species of domesticated and wild animals can serve as reservoir hosts for this parasite^27^. The two-host model^19^ that only includes bovine and human ignores other definitive hosts (e.g., pig, mouse). When bovine was no longer a major reservoir in endemic regions, Zhou *et al*.^9^ found that the two-host model did not fit the 5-year field observations well. Therefore, the two-host model might no longer reflect the dynamic transmission of *S. japonicum* when the schistosomiasis transmission is in the phase of elimination. The multi-host model includes other definitive hosts (e.g., pig, sheep/goat) in addition to bovine and humans^33^. Compared with bovines, other definitive hosts play a smaller role in the transmission of *S. japonicum*, and are often neglected. However, they become increasingly important in the elimination stage of this parasite in China^7^. We validated the multi-host model with more than 8-years longitudinal epidemiological data from the three study regions (i.e., the village of Guifang in Anhui province, the village of Changjiang in Hunan province and the town of Tezi in Sichuan province). The multi-host model fits the data well and the modeled prevalences were close to the adjusted prevalences. These indicate that our multi-host model can be used for assessing and predicting the effectiveness of different interventions on the transmission dynamics of *S. japonicum*, especially in the final stage of disease elimination in China.

The integrated control strategy (Scenario A), including human chemotherapy, bovine removal, improvement in sanitation, health education, and environmental modification, had been implemented in the village of Guifang, Anhui province since 2006, The (multi-host) modeled and the adjusted prevalences showed a good consistency (Fig. 3), and the results indicated that the integrated control strategy (Scenario A) was effective, but was not hugely successful since the prevalence of human infection was still as high as 3% after the 10-year interventions (Fig. 1). The two-host model provided no good fit^9^. Our findings from both the field observations and the multi-host model suggest that the control strategy (Scenario A) needs to be improved to eliminate the disease in the region^7^,^9^. Hence, most of snail habitats of the village were modified by agriculture practice (e.g., some crops like wheat and rape were planted in the snail habitats) since 2010 due to flooding control resulted from the Three Gorges Dam^34^. The prevalence of human infection continued to decrease and reduced to 1% in 2013. For Scenario B, the multi-host model also fit the field observations well (Fig. 1), and indicated a continuous decrease in the prevalence of human infection in future years. Using the routine interventions (scenario C in Fig. 2), the village of Changjiang in Hunan province had the prevalence of human infection, modeled or adjusted, of approximately 6%. Our findings from both the field observations and the multi-host model suggested that establishing safe grazing pastures and fencing pastures (scenario D in Fig. 4) were not effective. It is likely due to the difficulty of completely fencing pastures in the large marshland areas and maintaining pastures fenced for a long period of time^7^,^35^. Hence, in order to reduce the risk of human infection and interrupt the transmission of *S. japonicum*, the control strategy implemented in Junshan district was adjusted in 2013, and included bovine removal. There was a sharp decrease in the prevalence of human infection from 2012 to 2014 (Fig. 2). The multi-host model indicated a continuous decrease in the risk of human infection in future years (Fig. 2).

Human infections and density of living snails showed a somewhat complex relationship. There was a significant correlation of human infection with snail density in the village of Guifang village and the town of Tezi, but not at the village of Changjiang. When the snail density reduced from 19.93 snails/0.11 m^2^ in 2007 to 8.61 snails/0.11 m^2^ in 2009 however, the prevalence of human infection showed no obvious change in the village of Guifang with the snail density (Fig. 1). A similar phenomena also occurred in the village of Changjiang (Fig. 2). To determine the threshold of living snail density for blocking the transmission of *S. japonicum* therefore is very important for developing proper interventions. We used our multi-host model and field observations to explore the relationship of human infections with the density of living snails, and found that there might exit a threshold of living snail density for blocking the transmission of *S. japonicum*. Below the threshold value, the prevalence of human infection showed a decreasing secular trend even without any interventions, while above the threshold value the prevalence showed an increasing secular trend when there was no sufficient intervention (Fig. 4). Most of the estimated thresholds values were very low (Fig. 5) with their 50^th^ and 75^th^ percentiles being 0.009 and 0.028 snails/0.11 m^2^, respectively. These findings might partly explain the difficulties to interrupt schistosomiasis transmission in the remaining seven endemic provinces (Hubei, Hunan, Anhui, Jiangxi, Jiangsu, Sichuan and Yunnan provinces) in China^10^, especially in the lake and marshland regions with large areas of snail habitats and frequent flooding. Since the Three Gorges Dam was built in 2003. flooding has rarely occurred^34^. The reduction in density of snails in the village of Changjiang, to a large extent, was attributable to the declined water level and the snail-harboring marshlands are much less likely to be flooded, while *Oncomelania* snails have to live in water during their early stage of development^36^. Although the densities of living snails in our three study fields had decreased, they were still above the threshold values (Guifang; 0.106 vs. 0.0275 snails/0.11 m^2^ in 2014; Tezi: 0.003 vs. 0.0005 snails/0.11 m^2^ in 2013). These findings suggested that, in order to eliminate this disease in these areas, the current control strategies should be maintained for a certain period of time (e.g., more than 6 years) or be improved with some new sensitive diagnostic tools (e.g., rSP13-ELISA method, loop-mediated isothermal amplification) being used to find more patients infected with *S. japonicum* for treatment and with snails being more closely monitored^37^,^38^.

Our results demonstrated that the estimated thresholds varied substantially from one area to another, i.e., from 0.0005 snails/0.11 m^2^ in Puge county, Sichuan province to 0.12 snails /0.11 m^2^ in Jiangling county, Hubei province; however, half of these estimated thresholds were less than 0.009 snails/0.11 m^2^. One reason for the wide range might be related to the number of infection sources in endemic areas. For example, the estimated threshold value was relatively high (0.0275 snails/0.11 m^2^) and the density of bovines was relatively low (approximately 1 bovine/km^2^) at the village of Guifang in 2005, while the threshold value was relatively low (0.0005 snails/0.11 m^2^) and the density of bovines was high (approximately 29 bovines/km^2^) in the town of Tezi in 2005. Further studies are needed to understand the relationship between the number of infection sources and the threshold of living snail densities. Although the range of estimated threshold is wide, the transmission of *S. japonicum* might be interrupted in 75% of endemic regions when the density of living snails reduces to 0.006 snails/0.11 m^2^ (25^th^ percentiles).

Our study has some limitations. Intervention trials are needed to support our findings, which strongly suggest a threshold of living snail density for interrupting the transmission of *S. japonicum* in China. One of the Criteria for Control and Elimination of Schistosomiasis in China (GB15976-1995) for blocking the transmission of *S. japonicum* is that snails cannot be found for more than 1 year. Among 5 provinces (Shanghai, Fujian, Guangxi, Guangdong and Zhejiang) where the transmission of *S. japonicum* had been eliminated for approximately 20 years, snails are currently found in all these regions except for Guangdong province^39^. There was a possibility that the four provinces had very low snail densities rather than a total eradication of the snails. In addition, a number of assumptions were made for the multi-host model according to previous studies^9^,^1^9,^39^,^40^,^41^,^42^,^43^,^44^,^45^ and the field observations from the three study regions, caution should be taken when the model is applied to other circumstances.

## Conclusion

The national integrated strategy was effective for schistosomiasis control in the endemic areas, but it should be adjusted based on changes in human infection prevalence and/or environmental factors. There existed a threshold of living snail density for interrupting the transmission of *S. japonicum* that varied in specific areas. Our multi-host model and results could help people in a specific endemic area plan their own control strategies tailored to local situations to maximize control effects.

## Methods

### The mathematical model and parameters

**Figure 6:**
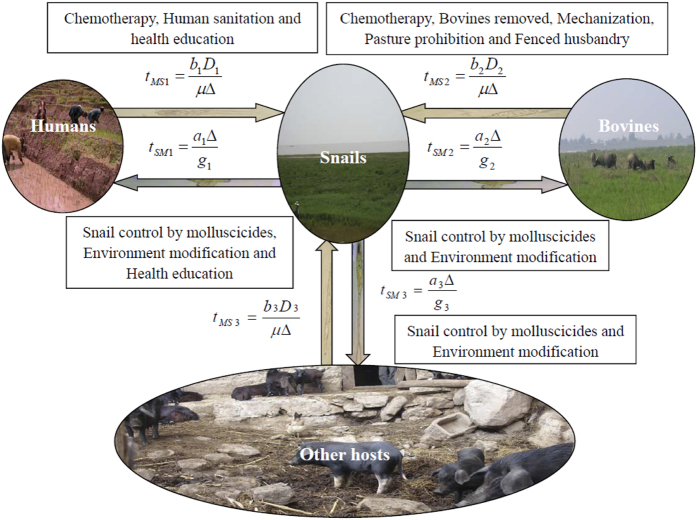
Pictorial pathways of schistosomiasis japonica transmission and control strategies in China.

Based on both the classic modeling framework (i.e., two-host model) by Barbour and an extended model by Williams and others, we proposed a multi-host model^33^. According to the distinctive patterns of exposure, disease course, and prevention/control strategies in China, the model is structured to track infections among definitive hosts, which are grouped into three separate classes (i.e. human, bovine, and other definitive hosts) (Fig. 6). Bovines are a major reservoir, therefore a major target for the disease prevention and control in China^7^. *Oncomelania* snail is the single intermediate host. This multi-host model is composed of a set of four differential equations, which track changes in prevalence in multiple definitive hosts (i.e., human, bovine and other definitive hosts) and in *Oncomelania* snails over time. The variables and parameters of this multi-host model were presented in Table 1.

Prevalence in humans:


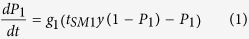


Prevalence in bovines:


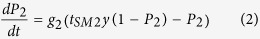


Prevalence in other definitive hosts (e.g. pig, goat, mice etc.):


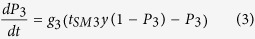


Prevalence in *Oncomelania* snails as an intermediate host:


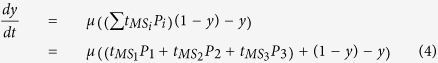


### Study areas

**Figure 7:**
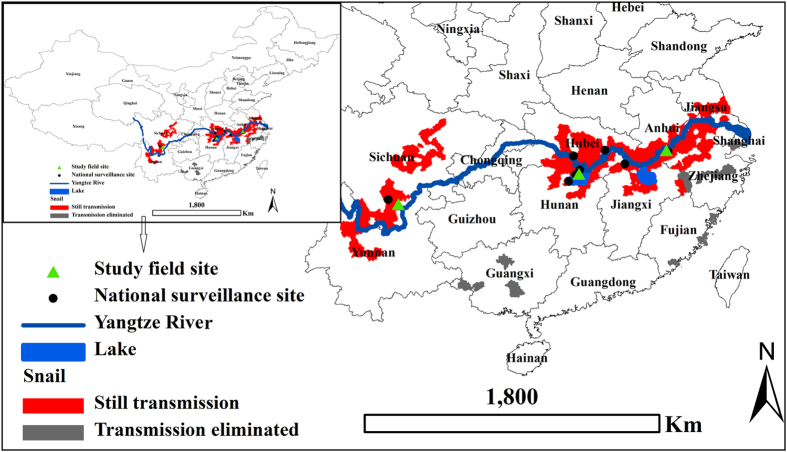
Location of the study sites, the national surveillance sites, China (ArcGIS10.0 (http://www.esri.com/software/arcgis/arcgis-for-desktop) was used to create the map, and the source of the map was bought by me).

This study were conducted in three most serious endemic areas of *S. japonica* in China historically, and they are 1) the Guifang administrative village, Guichi district, Anhui province; 2) Changjiang administrative village, Junshan district, Hunan province and 3) Tezi town, Puge County, Sichuan province (Fig. 7). The town of Tezi is relatively small with a population of approximately 4,000, and therefore the entire town was selected as a study site. The village of Guifang is located along the reach of the Qiupu River, which is a southern tributary of the Yangtze River, the longest river in Asia. A longitudinal investigation had been conducted at the village of Guifang from 2005 to 2014. The village of Changjiang is located along the reach of the Yangtze River and in the Dongting Lake (the second largest fresh water lake in China), and had a longitudinal investigation from 2007 to 2014. The town of Tezi lies in the northeast of Puge county, Sichuan Province, with an elevation ranging from 1,000 to 2,500 meters above the sea level, and has four administrative villages with a total area of 50.2 km^2^. There were about 8,000 livestock, including cattle, sheep, horses and pigs^46^. Tezi has a complex and varied topography with mountains and valleys being the most outstanding feature, representing a typical mountainous *S. japonicum*-endemic area in China. There was lack of lavatories, latrines or any other forms of sanitation facilities before 2005. A longitudinal investigation had been performed in the town from 2005 to 2013.

### Control measures

During the study periods, comprehensive control strategies had been carried out in the three study sites, and detailed information about the infrastructure and procedures of the control strategies has been provided elsewhere (Fig. 6)^9^,^46^,^47^. Except for routine interventions (i.e. chemotherapy, snail control by molluscicide, sanitation, environmental modification and health education), there were some additional different control measures implemented in the three study areas. Specifically, in the village of Guifang, all bovines were removed in February 2006 and most of the marshlands with snails had begun to be planted with wheat and rape by tractors since 2010. In the village of Changjiang, safe grazing pastures were established, and pasture in the snail-infested grasslands had been prohibited since 2010. All bovines were removed in May 2013. In the town of Tezi, it was very difficult or impossible to remove a very large amount of livestock including bovines that are the main source of household income for many residents^46^. Hence, pasture in the snail-infested grasslands was prohibited and safe grazing pastures had been established since 2006. Two hundred and ninety simple toilets had been constructed and 267 gas pools built since 2006. All snail habitats of the town had been treated by repeated mollusciciding and environmental management since 2008.

### Fecal samples and examinations

In each autumn during the study periods, all eligible participants aged 5 to 65 years were asked to provide a fecal specimen for stool examination by using the quantitative Kato-Katz thick smear technique (three slides)^9^. Residents with a positive fecal examination were treated with a single dose of praziquantel (40 mg/kg). Fecal samples were also collected from all bovines in these study areas every autumn of the study years and the infection status for *S. japonicum* was examined using the miracidial hatching test and a detailed procedure was described elsewhere^48^. These methods were carried out in accordance with the approved guidelines. Written informed consent was obtained from all adult participants and from the parents or legal guardians of children. Ethical approval for the study was obtained from corresponding village (local government), country (anti-schistosomiasis station), and provincial (schistosomiasis headquarters) authorities, and all experimental protocols were approved by the Ethical Review Committee of School of Public Health, Fudan University.

### Snail surveys

A survey of snail habitats was performed in the study sites in each autumn. The snail survey used the Chinese traditional method of random quadrant sampling: 0.11 m^2^ -sized frames, 20-m apart between frames in both villages of Guifang and Changjiang as lake and marshland regions, and 10-m apart between frames in the town of Tezi as a mountainous area^9^,^46^. All snails within the square frames were collected, counted and crushed to examine microscopically for schistosome infections.

### Data collected from the national surveillance system for schistosomiasis

The China’s national surveillance system for schistosomiasis was built in 1990. Since then, a longitudinal observation of the endemic situation of schistosomiasis japonica had been carried out at 13 national sentinel surveillance sites that represented different eco-epidemiological settings in China, and another surveillance site was added in 1995. In 2000, the national sentinel surveillance sites were further extended to 21 sites. Detailed information about the infrastructure and procedures of the national surveillance system and these surveillance sites has been provided elsewhere^49^,^50^. Specifically, residents aged 5–56 years in each surveillance site were checked for *S. japonicum* infection using serodiagnostic methods or/and Kato-Katz method. Bovines and other livestock (e.g. pigs, goats) were examined using the stool hatching method^48^. Snails were surveyed by using the Chinese traditional method of random quadrant sampling or an environmental sampling method in both spring and autumn each year^49^,^50^. In order to estimate the thresholds of snails below which the transmission of *S. japonicum* cannot be sustained, the data were only extracted from the surveillance sites where residents were only examined by using the Kato-Katz method (three slides) and the snail surveys were carried out by using the Chinese traditional method of random quadrant sampling^9^. The data included the prevalences of schistosomiasis infection in humans, bovines and other definitive hosts (e.g., pigs, goats). Data also included the schistosomiasis infection in snails and the density of living snails.

### Model assumptions

The transmission coefficients (*t*_*SM1*,_ *t*_*SM2*,_ *t*_*SM3*,_ *t*_*MS1*,_*t*_*MS2*_and *t*_*MS3*_) were estimated by substituting the endemic prevalences for humans, bovines, other definitive hosts and snails, and setting the four sets of differential equations above to 0^19^. Since the average lifespan of *S. japonicum* is approximately 4 years in humans, 1.5 years in bovines, 2.5 years in pigs, and 0.5 year in snails^9^,^19^,^40^, we assume that the duration of *S. japonicum* infection is 4 years for humans, 1.5 years for bovines, 2.5 years for other definitive hosts and 6 months for snails. The “observed” prevalence of human infection used in the multi-host model was adjusted according to the sensitivity of 65% and specificity of 100% for the Kato-Katz method (three slides) based on previous studies^9^,^39^,^41^. The unit of snail living density (snails/0.11 m^2^) was converted to snails/m^2^ in the model. Selective chemotherapy was carried out in the villages of Guifang and Changjiang that individuals with a positive fecal examination were treated with praziquantel. The sensitivity of Kato-Katz method was about 65%^39^,^41^ and certain residents with a positive examination were not treated due to travel or refused to take the medication, and therefore, we assumed the coverage of the human treatment with praziquantel being 45% with a success rate of 85% (e.g., 85% of treated infected humans were cured) in both the villages of Guifang and Changjiang in the basis of previous studies^9^,^19^,^44^.

In the village of Guifang village, there were important improvements in sanitation and health education during the study period. We assumed a 30% coverage of sanitation facilities and a 20% decrease in human water contact due to the sanitation improvement and health education. Based on the data on snail density and utilization of snail habitats (Fig. 1). There were an approximately 35% habitats of snails managed through agricultural practice with 40% reduction in snail density before 2010, and an approximately 80% snail habitats were managed through agricultural practice with 97% reduction in density of snails since 2010.

For the village of Changjiang, the coverage of selected treatment for bovines was similar to humans (i.e., the coverage was 45% and the drug efficacy was 85%) based on a previous study^45^. There were a 20% reduction in human water contact and a 30% coverage of sanitation facilities during the study period. Data on safe grazing pastures and fencing pastures in the village suggested a 10% reduction in bovine water contact and a 10% decrease in snail habitats contaminated by bovine stool during study period. Due to the decreasing water level since 2010, data on the snail density suggested a 75% snail habitats with 85% reduction in density of snails (Fig. 4).

For the town of Tezi, all residents aged 5–56 years and bovines with positive examination for infection were asked for taking praziquantel., Data indicated a coverage of mass treatment for both humans and bovines of 95%. A 20% reduction in human water contact and a 90% coverage of sanitation facilities were resulted from health education and sanitation improvement. A 10% decrease in bovine water contact and a 30% reduction in snail habitats contaminated by bovine stool were resulted from established safe grazing pastures and prohibiting pastures in the snail-infested grasslands during the study period. A 90% reduction in density of snails was assumed because of repeated mollusciciding and environmental management since 2008 in the bases of data on the snail density (Fig. 5).

### Statistical analysis and model simulation

All data were compiled in Microsoft Excel version 2013 (Microsoft Corp., Redmond, WA, USA). Statistical analyses were implemented using the SPSS (SPSS Inc., Chicago, IL, 2007). Control measures carried out in the three study fields were explicitly integrated in the multi-host model to predict the prevalence of human infection. The threshold value of snail density was estimated using the multi-host model based on data from the three study fields and the national schistosomiasis surveillance system. The numerical simulation was solved using Matlab software (The MathWorks, Inc., Natick, MA).

## Additional Information

**How to cite this article**: Zhou, Y.-B. *et al*. Multi-host model and threshold of intermediate host *Oncomelania* snail density for eliminating schistosomiasis transmission in China. *Sci. Rep.* **6**, 31089; doi: 10.1038/srep31089 (2016).

## Figures and Tables

**Table 1:** Description of variables and parameters for the multi-host model.

Symbol	Description
Human	
*P*_*1*_	Prevalence of *S. japonicum* infections in humans
*a*_*1*_	The rate at which a single person becomes infected when the density of infected snails is one per unit water area (m^2^)
*b*_*1*_	The rate at which an infected person causes snail infections
*D*_*1*_	Density of persons (per m^2^)
*g*_*1*_	Cure rate of man infected
*t*_*SM1*_	Snail to human transmission factor
*t*_*MS1*_	Human to snail transmission factor
Bovine	
*P*_*2*_	Prevalence of *S. japonicum* infections in bovines
*a*_*2*_	The rate at which a single bovine becomes infected when the density of infected snails is one per unit water area (m^2^)
*b*_*2*_	The rate at which an infected bovine causes snail infections
*D*_*2*_	Density of bovines (per m^2^)
*g*_*2*_	Cure rate of bovine infected
*t*_*SM2*_	Snail to bovine transmission factor
*t*_*MS2*_	bovine to snail transmission factor
Other definitive host	
*P*_*3*_	Prevalence of *S. japonicum* infections in other definitive hosts
*a*_*3*_	The rate at which a single other definitive host becomes infected when the density of infected snails is one per unit water area (m^2^)
*b*_*3*_	The rate at which a other infected definitive host causes snail infections
*D*_*3*_	Density of other definitive hosts (per m^2^)
*g*_*3*_	Cure rate of other definitive host infected
*t*_*SM3*_	Snail to other definitive host transmission factor
*t*_*MS3*_	Other definitive host to snail transmission factor
Snail	
Y	The proportion of snails infected
△	Density of living snails (per m^2^)
*μ*	Cure rate in snails
